# Supplementary figures and images for: Immobilization of Proteinase K for urine pretreatment to improve diagnostic accuracy of active tuberculosis
Source: PLoS One. 2021 Sep 21;16(9):e0257615. doi: 10.1371/journal.pone.0257615 (PMC8454978; doi:10.1371/journal.pone.0257615)

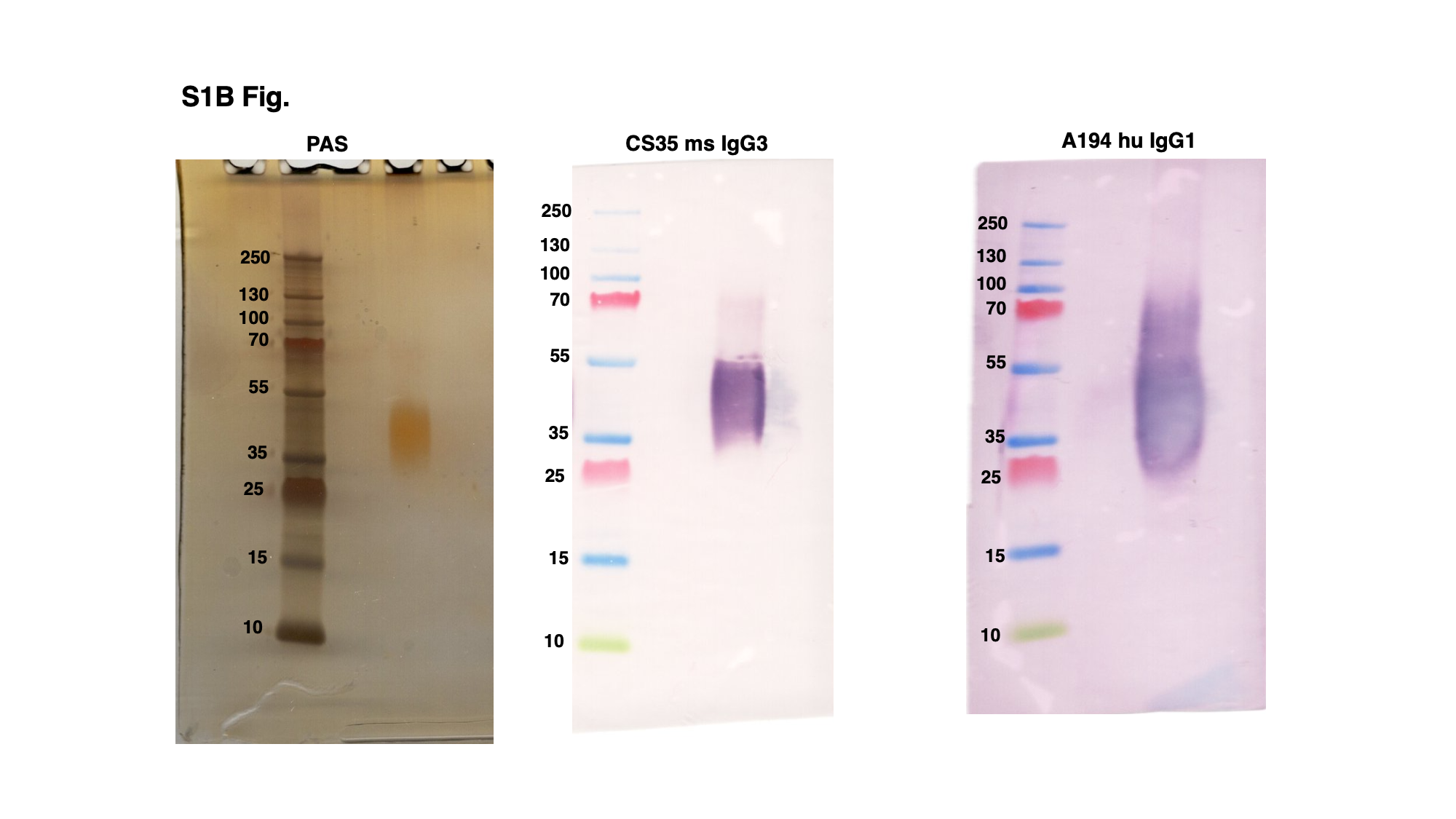

Supplement: S1 Fig — Original raw image uncropped SDS/PAGE and corresponding Western Blot. LAM purified from in vitro grown cells showing a tight smear (MW ~ 15–17 kDa). Western blot profile of the CDC1551 LAM with the anti-LAM mouse monoclonal CS35 antibody and anti-LAM human monoclonal A194, the two antibodies used as a pair in our Capture ELISA. (TIFF) [file pone.0257615.s001.tiff]
