# Supplementary material for: Immobilization of Proteinase K for urine pretreatment to improve diagnostic accuracy of active tuberculosis
Source: PLoS One. 2021 Sep 21;16(9):e0257615. doi: 10.1371/journal.pone.0257615 (PMC8454978; doi:10.1371/journal.pone.0257615)
Supplement: Attachment — Submitted filename: Response to Reviewers.docx [file pone.0257615.s002.docx]

August 13^th^, 2021

PONE-D-21-18899
Immobilization of Proteinase K for Urine Pretreatment to Improve Diagnostic Accuracy of Active Tuberculosis
PLOS ONE
.
To

Professor Seyed Ehtesham Hasnain
Academic Editor
PLOS ONE

Thank you for thoroughly reviewing our manuscript. Below, we have now addressed all your queries and listed these point-by point. All our responses (point by point) are hilited in red and copied below.

The two reviewers were appreciative of the work presented and had very constructive suggestions, which we addressed to the best of our ability. Original SDS/PAGE Western Blot without any edits has been included as a Supplementary material S1 Fig.

Figs 2 and 4 in the manuscript have been replaced with High Resolution Tif Figs. Most importantly. we have included a separate Ethics statement and revised some write ups in the Introduction and Methods and material section. Unfortunately, with me being the corresponding author, the style of writing repeats over time with the usage of similar statements. The edits are clearly visible in the version with tracking.

Hopefully the. resubmission will meet with the Journal style and goals.

Kind regards,

Delphi Chatterjee

Journal Requirements:

When submitting your revision, we need you to address these additional requirements.

1. Please ensure that your manuscript meets PLOS ONE's style requirements, including those for file naming. The PLOS ONE style templates can be found at 
<https://journals.plos.org/plosone/s/file?id=wjVg/PLOSOne_formatting_sample_main_body.pdf> and 
<https://journals.plos.org/plosone/s/file?id=ba62/PLOSOne_formatting_sample_title_authors_affiliations.pdf>

Level 1 subheadings corrected in the resubmission

2. You indicated that you had ethical approval for your study. In your Methods section, please ensure you have also stated whether you obtained consent from the patients included in the study or whether the research ethics committee or IRB specifically waived the need for their consent.

Ethics Statement is elaborated and added in the Methods and material section. Consent forms were distributed by FIND at the time of sample collection in ~2010.

3. We note that you have stated that you will provide repository information for your data at acceptance. Should your manuscript be accepted for publication, we will hold it until you provide the relevant accession numbers or DOIs necessary to access your data. If you wish to make changes to your Data Availability statement, please describe these changes in your cover letter and we will update your Data Availability statement to reflect the information you provide.

This was an oversight on my part. All our data (clinical details of samples and assay performance) are all included in the manuscript . There are no additional data to provide. We have added a statement as

**Data Availability**

All relevant data are presented within the paper.

4. PLOS ONE now requires that authors provide the original uncropped and unadjusted images underlying all blot or gel results reported in a submission’s figures or Supporting Information files. This policy and the journal’s other requirements for blot/gel reporting and figure preparation are described in detail at <https://journals.plos.org/plosone/s/figures#loc-blot-and-gel-reporting-requirements> and <https://journals.plos.org/plosone/s/figures#loc-preparing-figures-from-image-files>. When you submit your revised manuscript, please ensure that your figures adhere fully to these guidelines and provide the original underlying images for all blot or gel data reported in your submission. See the following link for instructions on providing the original image data: <https://journals.plos.org/plosone/s/figures#loc-original-images-for-blots-and-gels>. 
  
In your cover letter, please note whether your blot/gel image data are in Supporting Information or posted at a public data repository, provide the repository URL if relevant, and provide specific details as to which raw blot/gel images, if any, are not available. Email us at [plosone@plos.org](mailto:plosone@plos.org) if you have any questions.

There is only one SDS/PAGE Western Blot in the manuscript that is Fig 1B. We have now included the original run in the Supporting Information as **S1B Fig.** with same Fig Legend.

5. We noticed you have some minor occurrence of overlapping text with the following previous publication, which needs to be addressed:

- <https://www.sciencedirect.com/science/article/abs/pii/S1472979218300842?via%3Dihub>

In your revision ensure you cite all your sources (including your own works), and quote or rephrase any duplicated text outside the methods section. Further consideration is dependent on these concerns being addressed.

We have rephrased and cited sections that were similar to the above mentioned paper to the best of our knowledge. The clinical sample cohort section in the Methods and material has been rewritten and indicated in the tracks. The Ab section has also been rephrased

Additional Editor Comments:

Major Revision

[Note: HTML markup is below. Please do not edit.]

Reviewers' comments:

Reviewer's Responses to Questions

5. Review Comments to the Author

Reviewer #1: Comments:
Latent and disseminated TB is still big problem in diagnosis as well as in treatment regimen. In case if smear negative and culture negative TB-HIV case especially in comorbid situation this assay may have great advantages at POCT lavel. Present study of Panraksa, et al. was very nicely done using all methodological aspect to find out the better outcome in TB diagnosis with high sensitivity and specificity. Following suggestions may make more impact on this article:
Thank you for your encouraging comments
Abstract:
• Abbreviation must require for TB and Mtb upon first appearance in the text.
Corrected and changed accordingly
Method:
• Advise to put this section before Result and Discussion.- Done in the resubmitted version
• Ethical statement is missing. Should write in sub-heading in method section only.-Done and introduced Ethics Statement as a sub heading in Methods and material section see p. 7
• Provide figure in high resolution (Fig 2, Fig 4).

Replaced Fig 2 and 4 with High Resolution tif files. We have also used PACE per PLOS ONE guidance to check the submitted Figs quality. Fig 2 has been redrawn.

Results:
• Conclusion part is missing

Conclusion section is now added, see p 18.

Reviewer #2: I would like to appreciate efforts of the authors to improve Lipoarabinomannan (LAM) assay for the diagnosis of TB. It is beneficial as it is done in non invasive samples (urine) and it should be promoted with improved sensitivity using various pretreatment methods as done in this study.

Thank you

6. PLOS authors have the option to publish the peer review history of their article ([what does this mean?](https://nam10.safelinks.protection.outlook.com/?url=https%3A%2F%2Fjournals.plos.org%2Fplosone%2Fs%2Feditorial-and-peer-review-process%23loc-peer-review-history&data=04%7C01%7Cdelphi.chatterjee%40colostate.edu%7C4173e38d711a463bfe3808d952101895%7Cafb58802ff7a4bb1ab21367ff2ecfc8b%7C0%7C0%7C637631052610201468%7CUnknown%7CTWFpbGZsb3d8eyJWIjoiMC4wLjAwMDAiLCJQIjoiV2luMzIiLCJBTiI6Ik1haWwiLCJXVCI6Mn0%3D%7C1000&sdata=YR7ogzxOHtP7W7mAyzmyANkgFehON9sSxcMvcDwUa28%3D&reserved=0)). If published, this will include your full peer review and any attached files.


If you choose “no”, your identity will remain anonymous but your review may still be made public.


**Do you want your identity to be public for this peer review?** For information about this choice, including consent withdrawal, please see our [Privacy Policy](https://nam10.safelinks.protection.outlook.com/?url=https%3A%2F%2Fwww.plos.org%2Fprivacy-policy&data=04%7C01%7Cdelphi.chatterjee%40colostate.edu%7C4173e38d711a463bfe3808d952101895%7Cafb58802ff7a4bb1ab21367ff2ecfc8b%7C0%7C0%7C637631052610201468%7CUnknown%7CTWFpbGZsb3d8eyJWIjoiMC4wLjAwMDAiLCJQIjoiV2luMzIiLCJBTiI6Ik1haWwiLCJXVCI6Mn0%3D%7C1000&sdata=hA10n4MS8y3vtsTihZg43iLtNEq%2BQBsUw6YUe2IgdKw%3D&reserved=0).

Reviewer #1: No

Reviewer #2: No

  [NOTE: If reviewer comments were submitted as an attachment file, they will be attached to this email and accessible via the submission site. Please log into your account, locate the manuscript record, and check for the action link "View Attachments". If this link does not appear, there are no attachment files.]

While revising your submission, please upload your figure files to the Preflight Analysis and Conversion Engine (PACE) digital diagnostic tool, [https://pacev2.apexcovantage.com/](https://nam10.safelinks.protection.outlook.com/?url=https%3A%2F%2Fpacev2.apexcovantage.com%2F&data=04%7C01%7Cdelphi.chatterjee%40colostate.edu%7C4173e38d711a463bfe3808d952101895%7Cafb58802ff7a4bb1ab21367ff2ecfc8b%7C0%7C0%7C637631052610211457%7CUnknown%7CTWFpbGZsb3d8eyJWIjoiMC4wLjAwMDAiLCJQIjoiV2luMzIiLCJBTiI6Ik1haWwiLCJXVCI6Mn0%3D%7C1000&sdata=R%2B4RXnuf140z2030%2BKnQeC5QlqFvdis%2B%2BVJsLvhCtpc%3D&reserved=0). PACE helps ensure that figures meet PLOS requirements. To use PACE, you must first register as a user. Registration is free. Then, login and navigate to the UPLOAD tab, where you will find detailed instructions on how to use the tool. If you encounter any issues or have any questions when using PACE, please email PLOS at [figures@plos.org](mailto:figures@plos.org). Please note that Supporting Information files do not need this step.

*In compliance with data protection regulations, you may request that we remove your personal registration details at any time.*  [*(Remove my information/details)*](https://nam10.safelinks.protection.outlook.com/?url=https%3A%2F%2Fwww.editorialmanager.com%2Fpone%2Flogin.asp%3Fa%3Dr&data=04%7C01%7Cdelphi.chatterjee%40colostate.edu%7C4173e38d711a463bfe3808d952101895%7Cafb58802ff7a4bb1ab21367ff2ecfc8b%7C0%7C0%7C637631052610221460%7CUnknown%7CTWFpbGZsb3d8eyJWIjoiMC4wLjAwMDAiLCJQIjoiV2luMzIiLCJBTiI6Ik1haWwiLCJXVCI6Mn0%3D%7C1000&sdata=2Ya4uXzIIS86oURQavvGsvAkGfXbHZQB271p2aCExoU%3D&reserved=0)*. Please contact the publication office if you have any questions.*
